# Death of Dr. Thomas C. Brinsmade of Troy, N. Y.

**Published:** 1868-06

**Authors:** 


Death of Dr. Thomas C. Brinsmade of Troy, N. Y.—We are pained to learn of the death of Dr. Thomas C. Brinsmade, one of the most widely known and highly esteemed members of the profession. He died suddenly June 22d, aged 65 years, of disease of the heart, while presiding at a public meeting called in aid of the Rensselaer Institute. He has held many offices of trust and honor; was Vice President of the American Medical Association, President of the State Medical Society in 1857, and was one of the delegates to the Paris Scientific Congress in 1867. He was held in highest respect by the profession and the community in which he lived and labored, and his death will be widely felt and deeply mourned by all who knew him.

